# Supplementary material for: A Study on the Characteristics of Children’s Natural Activities in the Neighborhood and Their Influencing Factors: Evidence from Hangzhou, China
Source: Int J Environ Res Public Health. 2022 Dec 1;19(23):16087. doi: 10.3390/ijerph192316087 (PMC9738215; doi:10.3390/ijerph192316087)
Supplement: Supplementary file 1 [file ijerph-19-16087-s001.zip › ijerph-2055727-supplementary.pdf]

**File S1. Questionnaire**

Questionnaire number ___________ time ____________ location ___________________

**Children's Questionnaire**

**Dear children.**

Thank you very much for taking your valuable time to fill out this questionnaire carefully! We are graduate students from Zhejiang Agriculture and Forestry University. This research is to understand your natural activities and attitudes towards nature in detail, and aims to serve the child-friendly planning and design of outdoor spaces. The team promises never to divulge your information. Thank you!!!

**I. Basic Information**

1. Gender: □ boy □ girl
2. Age: ___________
3. Place of residence (neighborhood or street): ________________________
4. Do I have brothers and sisters □Yes □No

**II. Neighborhood natural activities**

1. In the past month (nearly 30 days), how many times have I been active in the outdoor natural space (park, woods, waterfront, garden, etc.) near my home? □0 times □1–2 times □3–5 times □6–8 times □8 times or more
2. What is the approximate duration of time I spend outdoors in natural spaces (parks, woods, water, gardens, etc.) each time?
□ 30 minutes or less □ 30 minutes-1 hour □ 1–2 hours □ 2–3 hours □ more than 3 hours
3. The neighborhood outdoor nature space I visit most often is _____________________________________

**III. Nature connectedness (Tick the box to indicate yes)**

1. I think it's great to get up early and watch the sunrise. □
2. I like to walk in outdoor natural spaces. □
3. I prefer indoor sports to outdoor sports. □
4. It's fun to collect rocks and shells. □
5. Being outdoors in natural spaces makes me happy. □
6. I like to touch plants. □
7. I like to touch animals. □
8. It is important to me to take care of small animals. □
9. If a bee or butterfly is in my house, I would rather catch and release it than kill it. □
10. I would be sad to see a puppy hit by a car. □
11. The sound of robins distracts me. □
12. People cannot live in a world without plants and animals. □
13. Picking up the garbage on the ground can protect the environment. □
14. We protect animals because we humans are also part of nature and have the obligation to protect it. □
15. Humans can change nature at will. □

**IV. environmental knowledge**

1. How many legs does a spider have?
□ 8 □ 6 □ 4

2. What is the task of bees? (Multiple choice)
□ pollination □ weather forecaster □ mosquito catching □ ash cleaning □ nectar production

3. Please find the balsam fir tree in the following pictures!

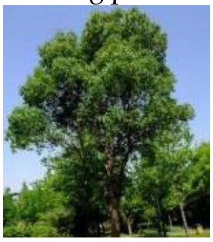

□

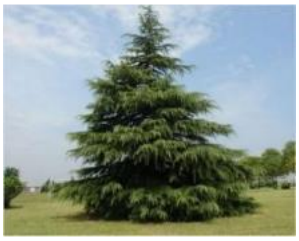

□

4. Please find the egrets in the pictures!

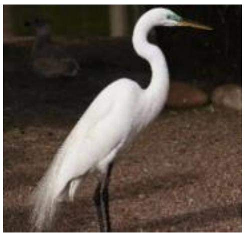

□

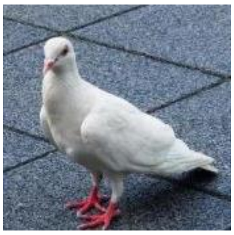

□

5. The rice we eat comes from? (Check the answer and find the corresponding two pictures)
□ Rice □ Wheat

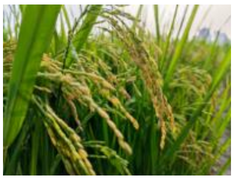

□

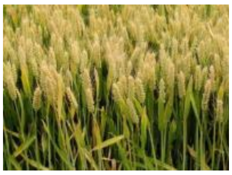

□

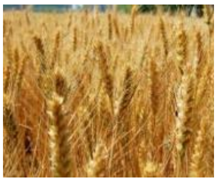

□

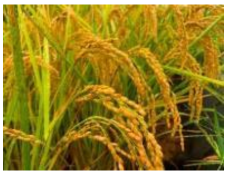

□

6. Which is the most environmentally friendly mode of transportation?
□ Airplane □ Train □ Car □ Bus □ Bicycle

7. Please mark on the horizontal line the serial number of the garbage corresponding to the garbage cans collected.
Garbage: 1 used batteries 2 banana peels 3 glass bottles 4 used newspapers 5 can boxes 6. discarded lamps

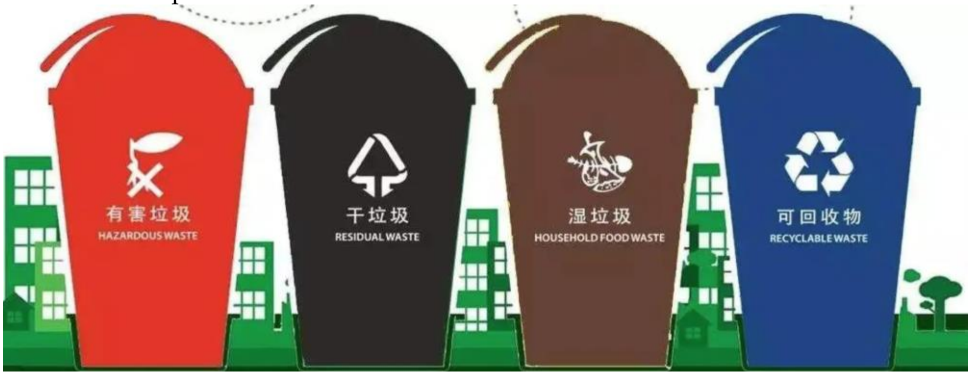


8. How does the water cycle work? Please write the term corresponding to the number in the box!
1 precipitation 2 flow into the ocean 3 surface outflow 4 condensation (cloud formation)
Evaporation -□-□-□-□

☺ Thank you!
